# Relationship between age, workplace slips and the effectiveness of slip-resistant footwear among healthcare workers

**DOI:** 10.1136/injuryprev-2022-044533

**Published:** 2022-04-12

**Authors:** Gillian Frost, Mark Liddle, Sarah Cockayne, Rachel Cunningham-Burley, Caroline Fairhurst, David J Torgerson, the SSHeW trial team, Sarah Cockayne

**Affiliations:** 1 HSE Science and Research Centre, Health and Safety Executive, Buxton, Derbyshire, UK; 2 York Trials Unit, University of York, York, North Yorkshire, UK

**Keywords:** Interventions, Occupational injury, Randomized Trial, Workplace, Older People

## Abstract

**Objectives:**

To explore any age-related trend in workplace slip rate and assess the effectiveness of appropriate slip-resistant footwear in preventing workplace slips by age.

**Methods:**

Secondary data analysis of the Stopping Slips among Healthcare Workers trial, a two-arm randomised controlled trial conducted between March 2017 and May 2019. 4553 National Health Service (NHS) staff across seven sites in England were randomised 1:1 to the intervention group (provision of 5* GRIP-rated slip-resistant footwear) or the control group (usual work footwear). The primary outcome was self-reported workplace slips, ascertained primarily through weekly text messages throughout the 14-week trial follow-up and analysed using mixed-effects negative binomial regression. This paper reports a control group-only analysis of the association between age and slip rate, and a full intention-to-treat analysis of the effectiveness of slip-resistant footwear by age.

**Results:**

The mean age of participants was 43 years (range 18–74). In the control group-only analysis, slip rate differed by age (p<0.001) with those aged 60+ having double the slip rate of those aged <30 years (95% CI 1.40 to 2.87). In the intention-to-treat analysis, the interaction between allocation and age was statistically significant (p=0.002). In addition, for all age groups except those aged <30 years, the slip rate in the intervention group was statistically significantly lower than the control group; the smallest incidence rate ratio (ie, the biggest effect) was 0.39 (95% CI 0.24 to 0.64) in the 60+ age group.

**Conclusion:**

The provision of appropriate slip-resistant footwear was more effective at reducing workplace slips for older NHS staff.

## Introduction

Slips, trips and falls on the same level are the most common workplace non-fatal injuries reported in Great Britain.^1^ A recent high-quality, large-scale randomised controlled trial (RCT), the Stopping Slips among Healthcare Workers (SSHeW) trial, found that the offer and provision of appropriate slip-resistant footwear reduced workplace slips by around 37% among National Health Service (NHS) staff in England.^2^ The workforce is getting older, and so it is important to understand how the effectiveness of any workplace intervention may differ by age.^3^ This brief report presents a secondary analysis of the SSHeW trial data to explore any age-related trend in workplace slip rate and whether the effectiveness of the provision of slip-resistant footwear in the workplace differed by age.

## Methods

Details of the SSHeW trial are reported elsewhere.^4^ Briefly, the SSHeW trial was a two-arm, parallel-group RCT conducted between March 2017 and May 2019 in seven NHS sites in England. NHS employees were eligible to participate if they were aged 18 years or older; adhered to a dress code policy; worked at least 22.5 hours a week on average; had a mobile phone and were willing to receive/send text messages; and worked in clinical areas, cafeterias, food preparation or service areas, or the general hospital environment. They were not eligible to participate if they were provided with footwear by their employer, agency staff or staff with less than 6 months remaining on their employment contract, or predominantly office or theatre based.

NHS staff were randomised 1:1 to the intervention or control group. The intervention was the offer and provision of five-star, GRIP-rated, slip-resistant footwear,^5^ with the control group asked to continue to wear their usual work footwear for the duration of the trial. The control group was offered a pair of the slip-resistant footwear at the end of the trial to aid retention.

The primary outcome measure was number of slips (no matter how minor) ascertained through weekly text messages over the 14-week trial follow-up. Where no text messages were received, the number of slips as reported on the final 14 week questionnaire was used instead. Statistical analysis of the primary outcome used intention to treat.^6^ To ensure comparability with the original analysis, similar methods were used in the current analysis. The 14-week slip rate was analysed using mixed-effects negative binomial regression. All regression models included gender, job role and pre-randomisation slip rate as fixed effects. NHS Trust was included as a random effect to account for potential clustering. The total number of hours worked over the 14-week trial period was the denominator for the rate.

The first step in this secondary data analysis was to adequately describe age in the model. This analysis was conducted using the control group only, since the effectiveness of the intervention may depend on age and hence distort any relationship between age and workplace slip rate. The model described earlier was repeated but additionally included age as a linear term, quadratic polynomial, cubic polynomial, non-linear effect using a cubic spline and a categorical variable (in 10-year age groups). The models were compared using the Akaike Information Criterion (AIC) and found that age as a categorical variable provided the best fit to the data (ie, it had the lowest AIC). As a sensitivity analysis, other variables measured at baseline, such as body mass index, were included as fixed effects if they were potentially confounding the association between age and slip rate; that is, they were associated with both age and slips (assessed using mixed-effects linear regression for age and mixed-effects negative binomial regression for slip rate, with random and fixed effects as specified previously, and p<0.05). To prevent overfitting, the number of potential confounders was reduced using a forward stepwise procedure (p<0.05 for inclusion and p>0.10 for exclusion) based on the same mixed-effects negative binomial regression for slip rate but excluding age. The age analysis was then repeated but additionally including this reduced list of potential confounders. The results were similar and made no difference to how age was best specified (results not shown).

An analysis looking at the effectiveness of the intervention by age was conducted on the full SSHeW trial population. The regression model was as specified earlier but also included group allocation, categorical age and the interaction between group allocation and age as fixed effects.

### Patient and public involvement

NHS staff (aged 20–71 years) from diverse roles including nurses, catering, housekeeping and doctors were consulted about the rationale for the trial, shoe styles, use of text messages, follow-up duration and testing slip resistance of usual work footwear. An NHS employee was a member of the joint Trial Steering Committee and Data Monitoring and Ethics Committee.

## Results

There were 4553 participants randomised between June 2017 and January 2019 (2275 intervention and 2278 control). Baseline characteristics were well balanced across groups. The mean age of the participants was 43 years (range 18–74), and most were female (85%) and worked in wards (54%), clinical areas (32%) or in the community (12%). After exclusions due to withdrawals and missing data, 4504 (2257 intervention, 2247 control) participants were included in the analysis. The full participant flow diagram^6^ and descriptions of all baseline variables^2^ are reported elsewhere.

Table 1 shows the number of people, number of working hours, crude incidence rates and adjusted incidence rate ratios (aIRRs) by age. In the control group-only analysis, there was strong evidence that slip rate differed by age (p<0.001). The slip rate for those aged 60 or older was statistically significantly greater than those for the other age groups (all p<0.05) and was double the slip rate at age <30 years (aIRR 2.00, 95% CI 1.40 to 2.87). The slip rate for those aged 40–49 years was also statistically significantly greater than the slip rate at age <30 years (aIRR 1.32, 95% CI 1.06 to 1.64), although this did not remain significant when adjusted for potential confounders (aIRR 1.22, 95% CI 0.98 to 1.52).

**Table 1:** Crude slip incidence rates by group allocation and incidence rate ratios for the intervention group compared with the control group by age

Age group (years)	Control group					Intervention group				Intervention relative to control group	
	N	Slips	Person-hours	Crude incidence rate* (95% CI)	aIRR† (95% CI)	N	Slips	Person- hours	Crude incidence rate* (95% CI)	aIRR‡ (95% CI)	P value
<30	378	721	169 091	0.160 (0.148 to 0.172)	1.00 (ref)	389	641	166 475	0.144 (0.133 to 0.156)	0.93 (0.73 to 1.20)	0.585
30–39	487	919	220 639	0.156 (0.146 to 0.167)	1.18 (0.94 to 1.48)	475	464	209 920	0.083 (0.076 to 0.091)	0.58 (0.46 to 0.74)	<0.001
40–49	632	1218	292 666	0.156 (0.147 to 0.165)	1.32 (1.06 to 1.64)	616	685	277 148	0.093 (0.086 to 0.100)	0.53 (0.43 to 0.64)	<0.001
50–59	633	1023	291 799	0.131 (0.124 to 0.140)	1.00 (0.80 to 1.25)	677	741	306 812	0.091 (0.084 to 0.097)	0.69 (0.57 to 0.85)	<0.001
60+	117	229	50 983	0.168 (0.147 to 0.192)	2.00 (1.40 to 2.87)	100	102	41 603	0.092 (0.075 to 0.112)	0.39 (0.24 to 0.64)	<0.001

*Per 37.5 person-hours (also known as one full-time working week).

†aIRRs comparing age groups among the control group only, estimated using mixed-effects negative binomial regression with sex, prerandomisation slip rate and job role as fixed effects, and site as random effect.

‡aIRRs comparing the intervention group to control group by age, estimated using mixed-effects negative binomial regression with sex, prerandomisation slip rate, job role, group allocation, and the interaction between group allocation and age as fixed effects, and site as random effect.

aIRR, adjusted incidence rate ratio; N, number of people; ref, reference category.

In the analysis looking at the effectiveness of the intervention, the interaction between allocation and age was statistically significant (p=0.002). For all age groups, the slip rate in the intervention group was lower than the control group, but this was not a statistically significant reduction when age was <30 years (aIRR 0.93, 95% CI 0.73 to 1.20). The reduction was statistically significant for all other ages (all p<0.001, table 1). The smallest aIRR of 0.39 (95% CI 0.24 to 0.64) was for the 60 and above age group and was statistically significantly smaller than the aIRR for the <30 and 50–59 age groups (p=0.002 and p=0.034, respectively); it was not statistically significantly different from the other age groups (both p>0.10).

## Discussion

This secondary analysis of data from the SSHeW trial found a statistically significant association between age and workplace slip rate, but there was no clear trend as age increased; for example, the slip rate did not monotonically increase with age. However, those aged 60 or over had double the workplace slip rate compared with those aged <30 years. An increase at older ages has been found in other studies, but the picture is far from consistent. A review by Chang *et al* 7 found that some studies reported an increase in occupational slips, trips and falls with age; some studies reported that younger workers had increased rates, and some studies did not find any significant relationship.

This analysis suggested that the intervention was least effective for participants aged under 30 years, and most effective for participants aged 60 years or over. This is an important result, given the context of an ageing workforce and provides reassurance that the intervention is still very effective among those at highest risk of slipping. The trial intervention was the offer and provision of slip-resistant footwear and, although participants were asked to wear the footwear all the time while at work, they were not mandated to do so. Differences in the intervention’s effectiveness might therefore be at least partially due to differences in compliance; for example, the styles may have been less appealing for younger workers.

The strengths and limitations of the SSHeW trial are discussed in detail elsewhere.^2 6^ Briefly, the main strengths of a robust methodology, large sample size and high engagement, generated high-quality data that can be used in secondary analyses such as that presented here. The limitations included the use of self-reported outcome data, where the participants could not be blinded to group allocation. In addition, some participants took longer than expected to collect the footwear, and they could also decide not to wear them. As described earlier, if this behaviour differed by age, then this could at least partially explain the difference in effectiveness observed here.

The SSHeW trial offered a valuable opportunity not only to look at the effectiveness of providing slip-resistant footwear by age but also to see how workplace slip rate varies by age. Older people tended to have more workplace slips, and the provision of slip-resistant footwear was more effective at these ages. As the workforce ages, slips will continue to be an important issue for workplaces, and provision of appropriate slip-resistant footwear can be a useful preventative measure in areas where it is not possible to prevent floor surfaces becoming slippery.

**Key messages**

**What is already known on the subject?**

- Appropriate slip-resistant footwear can reduce workplace slips by around 37% when offered and provided to National Health Service (NHS) staff in England, but whether this differs by age is unknown.

**What this study adds**

- NHS staff aged 60 or over tended to have more workplace slips compared with younger staff, and the provision of slip-resistant footwear was more effective at these ages with a 61% estimated reduction in slips for those aged 60 or over.

- As the workforce ages, slips will continue to be an important issue for workplaces and appropriate slip-resistant footwear can be a useful preventative measure in areas where it is not possible to prevent floor surfaces becoming slippery, especially for the older worker.
